# Leakage diffusion of underwater crude oil in wind fields

**DOI:** 10.1186/s40064-016-3457-x

**Published:** 2016-10-26

**Authors:** Liqiong Chen, Qi Liu, Yunyun Li, Rui Lu, Shijuan Wu, Xin Li, Tao Hou

**Affiliations:** 1 School of Petroleum Engineering, Southwest Petroleum University, Chengdu, Sichuan China; 2 China Petroleum Engineering Huabei Company, Renqiu, Hebei China; 3 National Computer Network Emergency Response Technical Team/Coordination Center of China, Beijing, China; 4 Guangxi Oil Production Plant, Southwest Oil and Gas Company, Sinopec, Deyang, Sichuan China; 5 China Petroleum and Natural Gas Co., Ltd. Da Gang Oil Field Branch, Tianjin, China

**Keywords:** Underwater, Crude oil pipe, Spilled oil, Diffusion, Numerical simulation

## Abstract

Leakage of underwater crude oil pipes causes severe pollution to soil and water, and results in great economic loss. To predict the diffusion area of spilled oil before it reaches the water’s surface and to reduce the time required for emergency response, numerical simulations were conducted on underwater spilled oil diffusion of bare crude oil pipes using FLUENT software. The influences of water-surface wind speed, leakage hole diameter, water velocity, and initial leakage velocity on oil diffusion were analyzed. The results revealed the following: (1) with wind blowing on the surface of the water, the vertical displacement of spilled oil jet-flow was affected by the combined action of water flow and wind, making it difficult for a high-speed jet-flow to form. A horizontal oil flow mostly moved in the direction of the bottom water, and frontier oil droplets dispersed quickly; (2) during the diffusion of spilled oil in water, the maximum horizontal displacement mostly increased linearly, while the maximum vertical displacement initially increased quickly and then slowed; (3) the greater the initial velocity and leakage hole diameter, the higher the oil jet-flow and the wider the diffusion area; the higher the water flow rate and water-surface wind speed, the smaller the vertical displacement of spilled oil. The existence of water-surface wind had no obvious influence on the horizontal displacement of underwater spilled oil.

## Background

With the ever-increasing transmission of crude oil, underwater crude oil pipeline projects are constantly underway. However, underwater leakage can cause severe soil and water pollution, which pose potential threats to the life of plants, animals, and humans as well as resulting in significant property loss (Yao 2013; Chet 1989). By investigating the leakage regularity of underwater crude oil pipes, the diffusion area of spilled oil can be predicted before it reaches the water’s surface, thereby reducing emergency response time (Wang 2006). Although crack leakages of submarine pipelines generally attract considerable attention, leakages of river-bottom pipelines garner little research concern (Gao et al. 2007; Fannelop 1972; Hoult and Fay 1970). Hence, it is necessary to put importance on the study of diffusion predictability of river-bottom crude oil pipelines.

In the last 20 years, diffusion models of underwater spilled oil have been developed based on three major technical approaches including theoretical study, model testing, and numerical simulation (Taylor and Grange 2002). In theoretical studies, fluid viscosity is rarely considered, resulting in considerable deviations between study results and practical results. A model test involves conducting experiments on scaled-down or full-scale models to obtain related data as well as detect design defects. Test results obtained this way are akin to practical conditions. However, the experimental devices are complicated and involve high costs, limiting this type of research. The numerical simulation method uses realistically constructed physical models to investigate engineering problems based on numerical calculations and image displays. In today’s world, with the rapid development of computers and software technology, using computer software to simulate engineering problems has become the leading trend.

In 1997, Yapa developed a 3D model of underwater spilled oil diffusion in the ocean, the results of which agreed well with the results of tests conducted in the Norwegian Sea. In 2006, Wang constructed a mathematical model of submarine pipeline leakage, which considered the influence of spilled oil emulsification on oil leakage. In 2008, Gao conducted simulations using FLUENT software and experimental validation of the flume and discovered the influence regularity of fluid velocity and manifold pressure on leakage. In 2011, Li and Ma established physical and mathematical 2D leakage diffusion models and studied variation regularities of spilled oil diffusing in soil and water as well as water flow velocity during oil leakage (Yapa and Li 1997; Wang et al. 2006; Shen et al. 1987; Gao 2008; Yapa and Chen 2004; Li et al. 2011; Li 2013).

In this paper, the influencing factors of underwater oil leakage from a cross-river pipeline in a wind field were analyzed and discussed using FLUENT software and preprocessing software GAMBIT. These research results can provide theoretical references for spilled oil recovery from cross-river pipeline leakages.

## Model establishment and analysis

In this study, a volume of fluid (VOF) model was adopted to solve the tracking problem of free interfaces. In the model, two or more types of fluids did not intersect with each other. For each additional item added to the model, a variable was correspondingly introduced to represent the phase volume fraction in the element. In the calculation element, the volume fraction of the *q*_*th*_ phase was set as *α*_*q*_. When *α*_*q*_ = 0, it was implied that the *q*_*th*_ fluid phase was empty in the element. When *α*_*q*_ = 1, it was implied that the *q*_*th*_ fluid phase was full in the element. For $0 < \alpha_{q} < 1$, there were interfaces between the *q*_*th*_ fluid phase and other multiphase fluids in the element. According to the distribution of volume fraction in a flow field, the curvature and shape of a free interface was calculated based on the volume fraction of the adjacent elements.

The volume fraction of the qth fluid phase can be expressed with Eq. (1).

1 $$\frac{{\partial \alpha_{q} }}{\partial t} + \overrightarrow {v} \cdot \nabla \alpha_{q} = 0$$

The corresponding control conditions are listed in Eq. (2).

2 $$\sum\limits_{q = 1} {\alpha_{q} } = 1$$

The densities at different locations in the flow field were different. In elements containing a mixture of several substances, Eq. (3) was utilized to calculate the related density in FLUENT, as follows.

3 $$\rho = \sum\limits_{q = 1}^{n} {\alpha_{q} \rho_{q} } ,\quad q = 1,2, \ldots ,n$$

where *α*_*q*_ is the volume fraction of the *q*_*th*_ phase; *ρ*_*q*_ is the density of the *q*_*th*_ phase; *t* is time; and $\overrightarrow {v}$ is the average velocity vector of the element.

In the diffusion model of a bare pipe in a wind field, both water and oil phases existed. Subscripts were used to represent different phases to track the volume fraction of the oil. The density of each element was expressed as the following:

4 $$\rho = \alpha_{\text{oil}} \rho_{\text{oil}} + (1 - \alpha_{\text{oil}} )\rho_{\text{water}}$$

The VOF model simulated two immiscible fluids by solving independent momentum equations and processing the volume percentage of each fluid flowing through. The momentum equation is shown in Eq. 5.

5 $$\frac{\partial }{\partial t}(\rho \overrightarrow {v} ) + \nabla (\rho \cdot \overrightarrow {v} \cdot \overrightarrow {v} ) = - \nabla P + \nabla \cdot [\mu (\nabla \overrightarrow {v} + \nabla \overrightarrow {v} )] + \rho \overrightarrow {g} + \overrightarrow {F}$$

where ρ is the fluid density; t is time; $\overrightarrow {v}$ is the fluid velocity vector; P is the external pressure; μ is the dynamic viscosity of the mixed fluid, $\mu = \sum\nolimits_{k = 1}^{n} {\alpha_{k} \mu_{k} }$; $\overrightarrow {g}$ is the acceleration of gravity; and $\overrightarrow {F}$ is the volume force.

The turbulent kinetic energy equation k in the leakage equation (standard *k*–*ε* transport equation) is shown in Eq. (6), and the diffusion equation ε is shown in Eq. (7).

6 $$\frac{\partial (\rho k)}{\partial t} + \frac{{\partial (\rho k\mu_{i} )}}{{\partial x_{i} }} = \frac{\partial }{{\partial x_{j} }}\left[ {\left( {\mu + \frac{{\mu_{t} }}{{\sigma_{k} }}} \right)\frac{\partial k}{{\partial x_{j} }}} \right] + G_{\text{k}} + G_{\text{b}} - \rho \varepsilon - Y_{\text{M}} + S_{\text{k}}$$

7 $$\frac{\partial (\rho \varepsilon )}{\partial t} + \frac{{\partial (\rho \varepsilon \mu_{i} )}}{{\partial x_{i} }} = \frac{\partial }{{\partial x_{j} }}\left[ {\left( {\mu + \frac{{\mu_{t} }}{{\sigma_{\varepsilon } }}} \right)\frac{\partial \varepsilon }{{\partial x_{j} }}} \right] + G_{1\varepsilon } \frac{\varepsilon }{k}(G_{\text{k}} + G_{3\varepsilon } G_{\text{b}} ) - G_{2\varepsilon } \rho \frac{{\varepsilon^{2} }}{k} + S_{\varepsilon }$$

where *k* is the turbulent kinetic equation of fluid; *μ* is the dynamic viscosity of fluid; *μ*_*t*_ is the turbulent velocity; *ε* is the turbulent dissipation rate; *μ*_*i*_ and *μ*_*j*_ are components of the turbulent viscosity; and *x*_*i*_ and *x*_*j*_ are coordinate components. *G*_*k*_ is the generation item of the turbulent power *k* caused by average velocity gradient; *G*_*b*_ is the generation item of the turbulent power *k* caused by buoyancy; *σ*_*k*_ and *σ*_*ε*_ are turbulent Prandtl constants of the *k* equation and *ε* equation, *σ*_*k*_ = 1.0 and *σ*_*ε*_ = 1.3. *Y*_*M*_ is the contribution of pulse expansion in the compressive turbulent; *C*_*1ε*_, *C*_*2ε*_, and *C*_*3ε*_ are constants, *C*_*1ε*_ = 1.44, *C*_*2ε*_ = 1.92 and *C*_*3ε*_ = 1. These three constants are empirical constants, which are measured by theoretical analysis and experiments; and *S*_*k*_ and *S*_*ε*_ are generalized source items defined by users.

The energy equation is shown in Eq. 8.

8 $$\frac{\partial }{\partial t}(\rho E) + \nabla \cdot \left[ {\vec{v}(\rho E + p)} \right] = \nabla \cdot (k_{\text{eff}} \nabla T) + S_{\text{h}}$$

where *ρ* is the fluid density; *E* is the fluid total energy; *P* is the external pressure; $\overrightarrow {v}$ is the fluid velocity vector; *T* is the fluid temperature; *k*_*eff*_ is the effective heat transfer coefficient; and *S*_*h*_ is the enthalpy source item of fluid.

## Simulation settings

Due to long-term flow scouring action during the operation of underwater oil pipelines, the pipes gradually become thin. Under the action of external force, holes occur in the pipes and oil spills out, driven by the constant pressure inside the pipes.

### Basic assumptions

1. This paper mainly focused on leakage diffusion of bare underwater pipes, while the diffusions of spilled oil in riverbed soil and on the water’s surface were not considered.
2. The leakage point was located right above the pipe, and the leakage hole was circular.
3. The oil spill was an isothermal and adiabatic process.
4. The oil volume fraction in the pipe was 1 (no gas existed), i.e. the leakage diffusion process became an oil–water double-phase flow problem.
5. The water-surface wind velocity and water flow velocity were constants.
6. Due to the presence of external pressure under the water, the leakage of oil is very difficult to observe if the leakage hole diameter is too small. Therefore, in this paper, we will mainly study the leakage condition of large aperture.

### Model construction

#### Basic parameters

The basic parameters of the simulation process are listed in Tables 1 and 2.

**Table 1:** Fluid physical parameters

Medium	Density (kg/m^3^)	Specific heat (J/kg K)	Kinematic viscosity (m^2^/s)	Temperature (K)	Thermal conductivity (W/m K)	Acceleration of gravity (m/s^2^)
Oil	860	–	2.4 × 10^−3^	298.15	–	9.8
Water	998.2	4182	1.003 × 10^−3^	298.15	0.6	9.8
Air	1.225	1006.43	1.7894 × 10^−5^	298.15	–	–

**Table 2:** Other parameters

Parameters	Water depth (m)	Air computing domain (m)	Wind speed (m/s)	Water flow velocity (m/s)	Leakage hole diameter (m)	Leakage initial velocity (m/s)
Value	10	1.0	6.0	7.0	0.1	20

#### Physical model

**Fig. 1:**
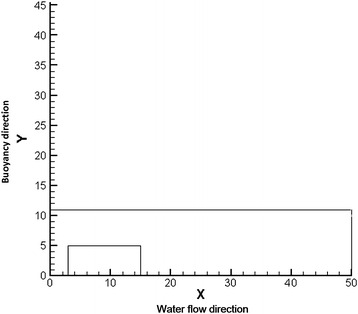
Physical model of diffusion of underwater pipe leakage in wind field

The existence of a wind field changed the diffusion path of the spilled oil by affecting the flow velocity of the surface water. In this study, a 2D water–air model was constructed. The water flow direction was set as the *X*-axis, and the buoyancy force direction of the river water was set as the *Y*-axis. The calculation range was 50 × 11 m^2^, in which the calculation region of the air was 1 × 50 m^2^. The physical model is shown in Fig. 1.

#### Simulation conditions

##### Mesh

**Fig. 2:**
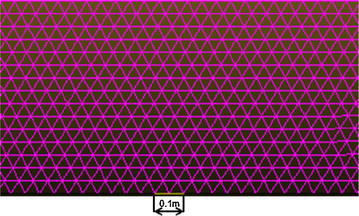
Locally refined elements above leakage hole

The area investigated was a 50 × 10 m^2^ rectangular area surrounded by the pipe wall and water flow zone. By assuming that the leakage hole was located right above the pipe, the calculation area was divided into two parts, one of which was a 10 × 5 m^2^ rectangular area above the leakage hole. Since this was close to the leakage hole, the mesh in this area was refined with Tri-type elements based on the Pave method. The second area was the water outside the rectangular area. This area was meshed with Quad-type elements based on the Map method. There were a total of 122,932 elements in the model (Fig. 2).

##### Boundary conditions

The water inlet, wind inlet, and oil leakage hole were set as velocity inlets, with velocities set at 7, 6, and 20 m/s, respectively. The velocity directions at the water and wind inlets were perpendicular to the left boundary of the model, while the velocity direction at the oil leakage hole was perpendicular to the bottom edge of the model. The water and wind outlets were set as pressure outlets with directions set as perpendicular to the right boundary of the model. The interface between the air and water was set as the interior, the interface between the refined area and the unrefined area was set as interfacial, and the remaining boundaries were set as walls.

##### Initial conditions

The basic phase was set as the air and the secondary phases were water and oil. The density of the basic phase was 1.225 kg/m^3^, the water density was 998.2 kg/m^3^, and the crude oil density was 860 kg/m^3^. At the initial moments, the water volume fraction below the free surface was set as one using the Patch function.

##### Solver condition

A PISO algorithm based on the pressure solution was adopted with the first-order upwind scheme and standard *k*–*ε* model selected. The state was set as unsteady. The calculation step was 0.05.

#### Preliminary simulation results

**Fig. 3:**
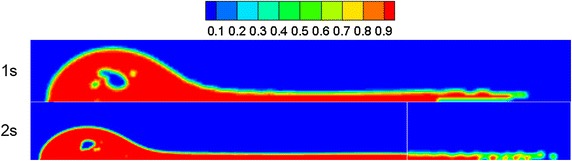
Diffusion range of spilled oil after 1 and 2 s of leakage

During calculations, when the water-surface wind velocity was 6 m/s, it was discovered that the flow field became steady at 1.0 s, and the oil was set to start leaking at this moment. Figure 3 shows the volume distribution of spilled oil after 1 and 2 s of leakage when the wind velocity was 6 m/s, water flow speed was 7 m/s, and the initial oil spilling speed was 20 m/s.

As shown in Fig. 3, it was indicated that under the combined influence of water flow and wind field, it was difficult for the spilled oil to form a relatively high jet-flow in a vertical direction. The spilled oil mostly gathered along the direction of the river-bottom water flow, and the frontier oil droplets dispersed very quickly in the water.

## Factors affecting underwater spilled oil diffusion

Leakage diffusion of underwater oil pipelines is affected by many external factors. To narrow these influences down, this paper focused on only four factors, including water-surface wind velocity, leakage hole diameter, water flow velocity, and initial oil leakage velocity. In this chapter, the parameters which is not as a variable all refer the data in Table 2.

### Water-surface wind velocity

**Fig. 4:**
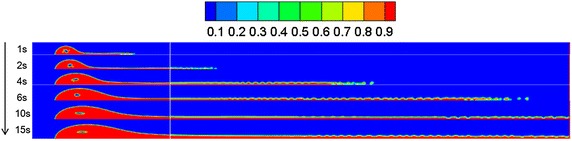
Volume distribution of spilled oil after different leakage durations with water-surface wind velocity of 6 m/s

**Fig. 5:**
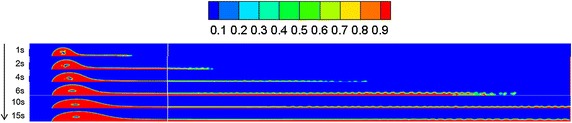
Volume distribution of spilled oil after different leakage durations with water-surface wind velocity of 2 m/s

**Fig. 6:**
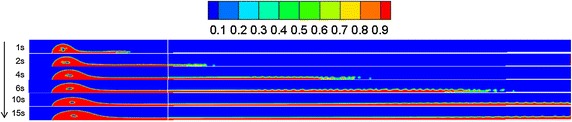
Volume distribution of spilled oil after different leakage durations with water-surface wind velocity of 0 m/s

Referring to “Preliminary simulation results” section, the diffusion behaviors of spilled oil under different wind velocities were compared. The volume distribution of spilled oil under different wind speeds and leakage durations are shown in Figs. 4, 5 and 6.

As shown in the images above, the spilled oil mostly diffused along the river-bottom water flow direction in a horizontal manner under the combined action of wind and water, and the frontier oil droplets were dispersed quickly by the water flow. The smaller the wind speed, the smaller the horizontal displacement of the spilled oil. In the vertical direction, oil columns formed around the leakage hole under the action of buoyancy.

**Fig. 7:**
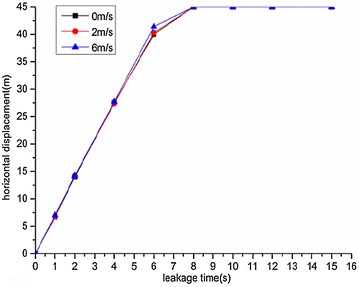
Variation of the maximum horizontal displacement *H*_*max*_ under different wind velocities

**Fig. 8:**
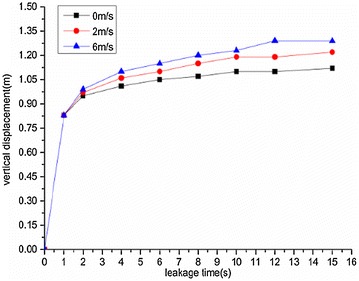
Variation of the maximum vertical displacement *V*_*max*_ under different wind velocities

The variation regularities of the maximum horizontal and vertical displacement of underwater oil diffusion are presented in Figs. 7 and 8, respectively.

The following conclusions can be drawn from Figs. 7 and 8:

1. The maximum diffusion displacements of the spilled oil in both the horizontal and vertical directions increased linearly under all wind velocity conditions. The reason for this phenomenon is that the existence of a wind field on the water’s surface can accelerated the leeward diffusion of oil films and the faster the wind speed is, the faster the oil film expands. Therefore, the vertical displacement of leakage oil will be reduced in the same time period.
2. The higher the water-surface wind velocity, the greater the maximum vertical displacement. The water-surface wind velocity had no significant influence on the horizontal displacement of the spilled oil.

### Leakage hole diameter

**Fig. 9:**
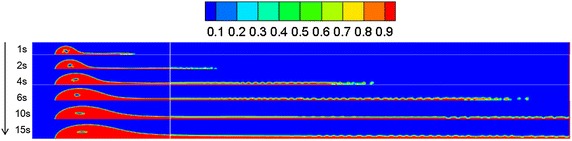
Volume distribution during oil diffusion after various durations with leakage hole diameter of 0.1 m

**Fig. 10:**
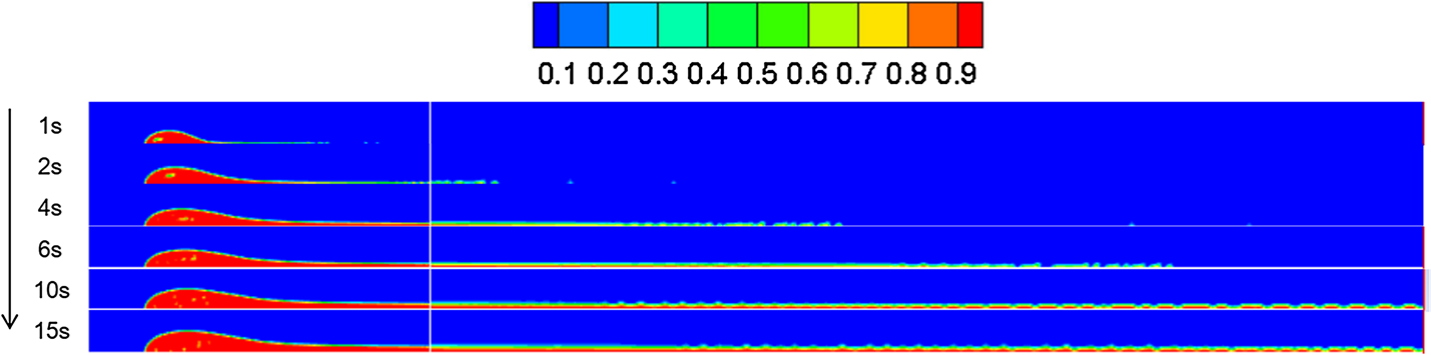
Volume distribution during oil diffusion after various durations with leakage hole diameter of 0.05 m

**Fig. 11:**
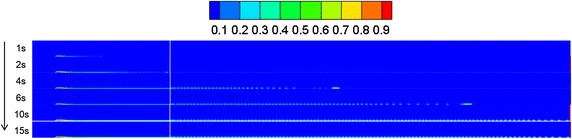
Volume distribution during oil diffusion after various durations with leakage hole diameter of 0.01 m

By referring to Sect. "Preliminary simulation results", a leakage diffusion model with hole diameters of 0.05 and 0.01 m was constructed. Volume distribution diagrams of oil diffusion under different leakage hole diameters and diffusion durations were compared to these results, as presented in Figs. 9, 10 and 11.

As the above images demonstrate, the greater the leakage hole diameter, the higher the leakage amount within the same period. In a wind field, the smaller the leakage hole, the more difficult the formation of a continuous jet-flow, and the easier the diffusion of the frontier oil straying from the main oil body. Under the disturbance of wind, spilled oil transformed into individual droplets and drifted with the wind. Under the condition of a leakage hole diameter of 0.01 m, the spilled oil usually diffused in the river-bottom along the flow direction, which was difficult to notice.

**Fig. 12:**
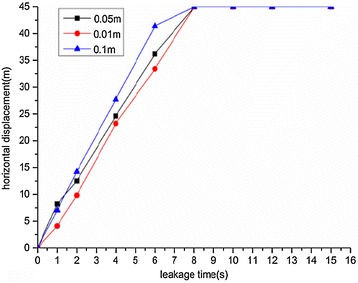
Variations of maximum horizontal displacement *H*_*max*_ during oil diffusion with different sizes of leakage holes

**Fig. 13:**
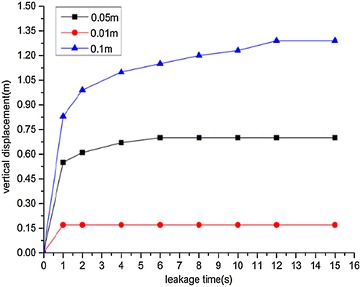
Variations of maximum vertical displacement *V*_*max*_ during oil diffusion with different sizes of leakage holes

Variation diagrams of the maximum horizontal and vertical displacements during underwater oil diffusion are presented in Figs. 12 and 13, respectively.

According to the above images, the following implications can be drawn:

1. With various leakage hole diameters, both the maximum horizontal and vertical displacements of the spilled oil followed linearly increasing tendencies. The greater the leakage hole diameter, the greater the diffusion displacement increase rate.
2. When the leakage hole diameter was 0.01 m, the vertical displacement of the spilled oil was small and remained almost constant during diffusion.

### Water flow velocity

**Fig. 14:**
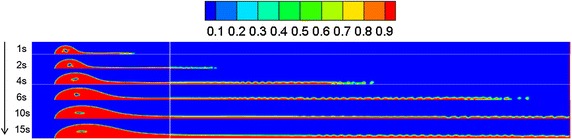
Volume distribution during oil diffusion in water flow velocity of 7 m/s

**Fig. 15:**
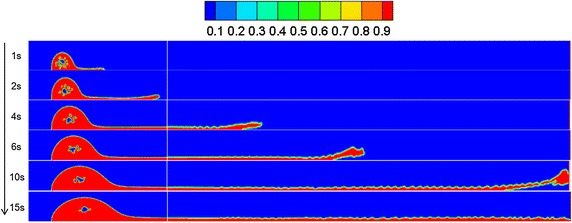
Volume distribution during oil diffusion in water flow velocity of 4 m/s

**Fig. 16:**
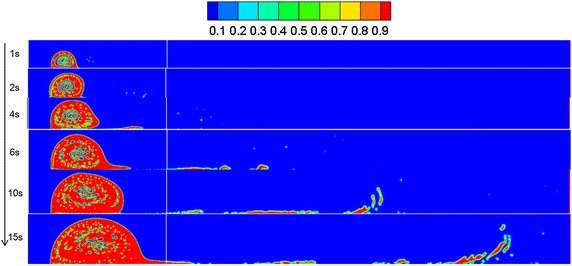
Volume distribution during oil diffusion in water flow velocity of 2 m/s

By referring to Sect. "Preliminary simulation results", the leakage behaviors under water flow velocities of 2, 4, and 7 m/s were compared, and the results of the volume diffusion under different water flow velocity and diffusion durations are displayed in Figs. 14, 15 and 16.

As displayed in the above images, the faster the water flow, the more difficult the formation of a continuous jet-flow, the lower the oil column, and the greater the horizontal displacement of the spilled oil. With a decrease of water flow velocity, the oil column height increased, and the frontier oil droplets presented an obvious increasing tendency under the action of buoyancy, i.e. the influence of water buoyancy force on the spilled oil became more obvious.

**Fig. 17:**
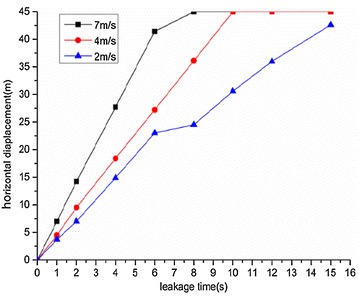
Variation of maximum horizontal displacement *H*_*max*_ during oil diffusion under different water flow velocities

**Fig. 18:**
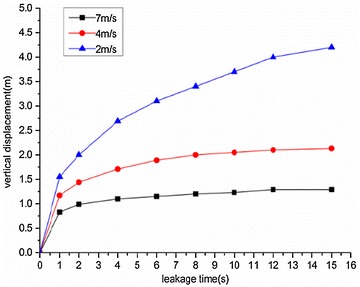
Variation of maximum vertical displacement *V*_*max*_ during oil diffusion under different water flow velocities

Variations of the maximum horizontal and vertical displacements during oil diffusion are presented in Figs. 17 and 18, respectively.

Figures 17 and 18 demonstrate the following conclusions:

1. Under different water flow velocities, the maximum value of the horizontal displacement of the spilled oil increased linearly. The faster the water flow, the more significant the horizontal displacement variation.
2. The growth of the vertical displacement could be divided into two parts. Within 1 s of diffusion, the vertical displacement increased sharply, but slowed after 1 s of diffusion. During a water flow velocity of 2 m/s, the vertical displacement increase was the fastest. The faster the water flow, the smaller the vertical displacement of the spilled oil.

### Leakage initial velocity

**Fig. 19:**
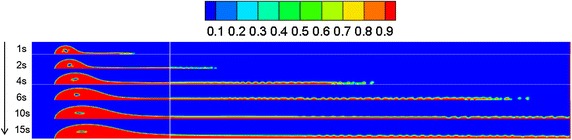
Volume distribution during oil diffusion with initial leakage velocity of 20 m/s

**Fig. 20:**
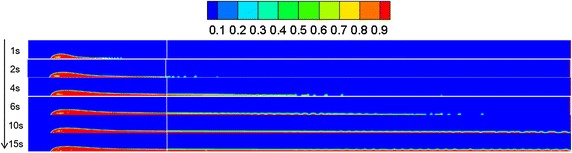
Volume distribution during oil diffusion with initial leakage velocity of 10 m/s

**Fig. 21:**
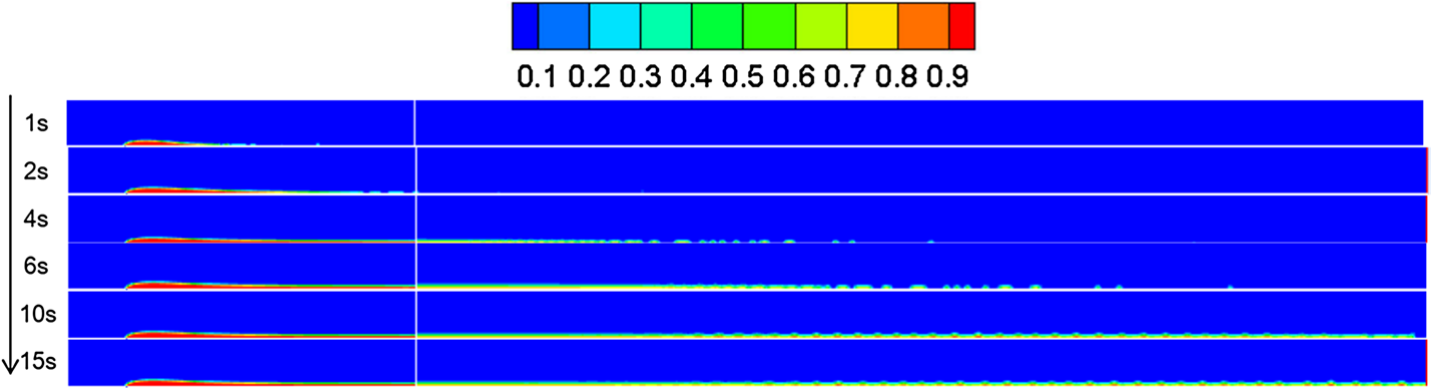
Volume distribution during oil diffusion with initial leakage velocity of 5 m/s

Based on “Preliminary simulation results” section, the initial leakage velocity of the oil was considered as a variable. By comparing the leakage conditions under various initial leakage velocities, the volume distribution diagrams under different leakage initial velocities and diffusion durations were obtained, as shown in Figs. 19, 20 and 21.

According to the above figures, when the initial leakage velocity was relatively low (i.e. 5 m/s), it was impossible for a jet-flow to form, and the spilled oil only settled along the horizontal direction. With an increase of leakage velocity, the oil gradually rose vertically under the combined influence of buoyancy and the wind field, and the diffusion frontier droplets demonstrated a distribution pattern.

**Fig. 22:**
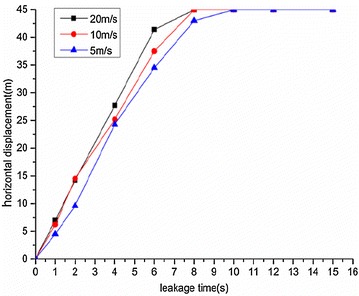
Variation of maximum horizontal displacement *H*_*max*_ during oil diffusion under different initial leakage velocities

**Fig. 23:**
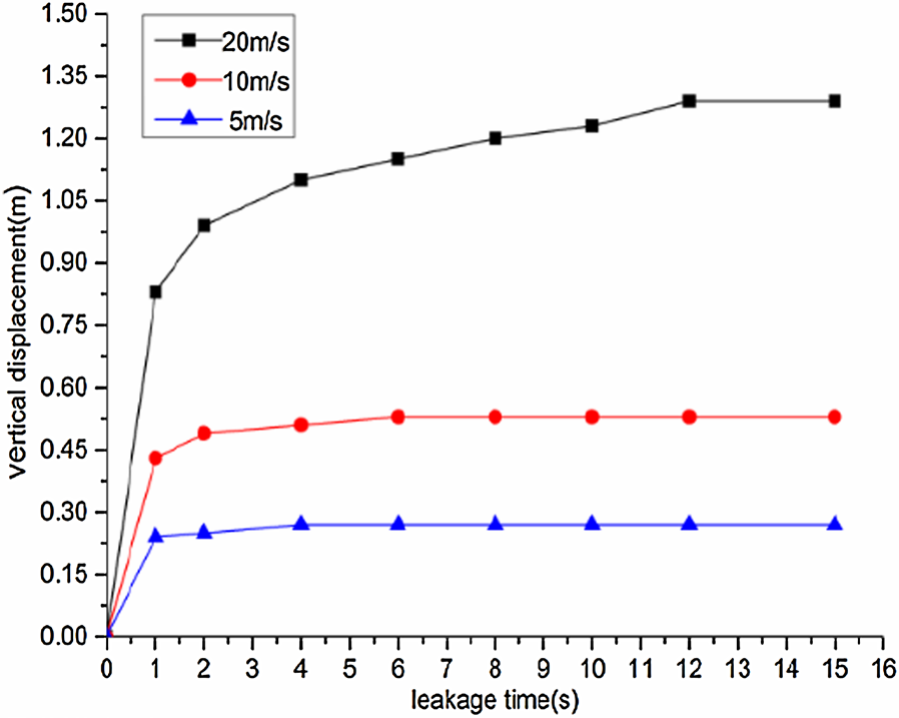
Variation of maximum vertical displacement *V*_*max*_ during oil diffusion under different initial leakage velocities

Variation regularities of maximum horizontal and vertical displacement during diffusion are shown in Figs. 22 and 23, respectively.

According to the above figures, the following conclusions could be drawn:

1. When the initial leakage velocity was different, the maximum horizontal displacement of the spilled oil increased linearly. The greater the initial leakage velocity, the larger the horizontal displacement increase rate.
2. The increase of vertical displacement was divided into two parts. Within 1 s of leakage, the vertical displacement increased quickly and then slowed. When the initial leakage velocity was relatively low (under 10 m/s), the vertical displacement of the spilled oil remained almost constant after 1 s.

## Conclusions

In this paper, FLUENT software was adopted to conduct numerical simulations of cross-river crude-oil pipeline leakage diffusion tendencies. A 2D underwater crude-oil diffusion model was created, and the influence of water-surface velocity, leakage hole diameter, water flow velocity, and initial leakage velocity on the diffusion range were investigated and compared. These research results can provide a scientific basis for the preparation of emergency response equipment in the future. Detailed analyzes and conclusions were summarized as follow:

1. The existence of a wind field on the water’s surface accelerated the leeward diffusion of oil films and the faster the wind speed is, the faster the oil films expands and the vertical displacement of leakage oil will be reduced in the same time period. Due to the combined influence of water flow and wind field on the vertical displacement of a spilled-oil jet-flow, the jet-flow height was limited. During leakage, the spilled oil usually flowed along the water flow direction on the river bottom; however, the frontier oil droplets easily dispersed and moved upward under the influence of buoyancy.
2. Factors affecting the diffusion of underwater spilled oil were analyzed, including water-surface wind velocity, leakage hole diameter, water flow velocity, and initial leakage velocity. During the diffusion of spilled oil in water, the maximum horizontal displacement increased linearly. The maximum vertical displacement increased quickly during the initial stage of leakage and slowed with continuous diffusion.
3. The greater the initial leakage velocity and leakage hole diameter, the higher the jet-flow and the wider the diffusion range. The greater the water flow velocity and water surface velocity, the smaller the vertical displacement of oil. The existence of a water-surface wind field showed no noticeable influence on the horizontal displacement of underwater spilled oil.

But there are still some shortcomings in this research process:

1. Oil spills may occur at any part of the pipe, this paper only considered the possibility of the occurrence of the oil spill over the pipeline.
2. This paper is only concerned with the most common circular hole leakage, for the other different shapes of the hole leakage is not considered, such as triangle, quadrilateral, etc.
